# Behavioural impairments in a mouse model of Kabuki syndrome associated with dopaminergic and neuroinflammatory modulations

**DOI:** 10.1017/neu.2025.17

**Published:** 2025-04-14

**Authors:** Thalles F. Biondi, Silvia M.G. Massironi, Eduardo F. Bondan, Thiago B. Kirsten

**Affiliations:** 1 Psychoneuroimmunology Laboratory, Program in Environmental and Experimental Pathology, Paulista University, São Paulo, Brazil; 2 Department of Pathology, School of Veterinary Medicine and Animal Science, University of São Paulo, Brazil

**Keywords:** Mutant mouse strain, grooming, stereotyped behaviour, dopamine, astrocytes, microglia

## Abstract

**Objective:**

Kabuki syndrome is a rare multisystem congenital disorder characterised by specific facial malformations and several other symptoms, including motor impairments, increased susceptibility to infections, immune mediators’ deficits, anxiety, and stereotyped behaviours. Considering the reports of motor impairments in Kabuki syndrome patients, the first hypothesis of the present study was that this motor dysfunction was a consequence of striatal dopaminergic modulation. The second hypothesis was that the peripheral immune system dysfunctions were a consequence of neuroinflammatory processes. To study these hypotheses, the mutant *bapa* mouse was used as it is a validated experimental model of Kabuki syndrome.

**Methods:**

Exploratory behaviour, anxiety-like behaviour (light-dark test), repetitive/stereotyped behaviour (spontaneous and induced self-grooming), and tyrosine hydroxylase (TH), astrocyte glial fibrillary acidic protein (GFAP), and ionised calcium-binding adaptor molecule 1 (Iba1) striatal expressions were evaluated in female adult *bapa* and control mice.

**Results:**

Female *bapa* mice did not present anxiety-like behaviour, but exploratory hyperactivity and stereotyped behaviour both on the spontaneous and induced self-grooming tests. Striatal TH, GFAP, and Iba1 expressions were also increased in *bapa* mice.

**Conclusion:**

The exploratory hyperactivity and the stereotyped behaviour occurred in detriment of the striatal dopaminergic system hyperactivity and a permanent neuroinflammatory process.

Significant outcomes

- *Bapa* mice presented exploratory hyperactivity and stereotyped behaviour. Striatal tyrosine hydroxylase, glial fibrillary acidic protein, and ionised calcium-binding adaptor molecule 1 expressions were also increased in *bapa* mice.
- The behavioural phenotype was a result of the dopaminergic system hyperactivity.
- Dopaminergic system modulation was associated with a neuroinflammatory process.

Limitations

- Molecular studies about dopamine, tyrosine hydroxylase, cytokine levels, and the neuroinflammatory pathway.

Highlights

- *Bapa* mice presented exploratory hyperactivity and stereotyped behaviour. Striatal tyrosine hydroxylase, glial fibrillary acidic protein, and ionised calcium-binding adaptor molecule 1 expressions were also increased in *bapa* mice.
- The behavioural phenotype was a result of the dopaminergic system hyperactivity.
- Dopaminergic system modulation was associated with a neuroinflammatory process.

## Introduction

Kabuki syndrome is a rare multisystem congenital disorder characterised by specific facial malformations (peculiar face with long or wide palpebral fissures, lower lateral eyelid eversion, arched eyebrows with the lateral third dispersed, prominent ears, depressed nasal tip, and skeletal and dermatoglyphic abnormalities) that resemble the stage makeup used in Kabuki, a Japanese traditional theatrical form (Boniel *et al*., 2021). Several other symptoms are documented, including poor physical growth, cardiac, gastrointestinal, and renal anomalies, motor and cognitive impairments, increased susceptibility to infections, and immune mediators’ deficits (Van Laarhoven *et al*., 2015; Wang *et al*., 2019). Anxiety (Kalinousky *et al*., 2022) and stereotyped behaviours (Sertcelik *et al*., 2016; Boniel *et al*., 2021) have been also reported in patients with Kabuki syndrome.

To study Kabuki syndrome and its behavioural, brain, immune, and genetic mechanisms, our group developed a mouse model: *bapa* mice. The *bapa* mouse model is a recessive mutant mouse also known as *bate palmas* (BALB/c^*bapa*^). It arose from N-ethyl-N-nitrosourea mutagenesis (Massironi *et al*., 2006). Genetic sequencing revealed the missense mutation NM_001033276:c.A3865G:p.T1289A in the lysine (K)-specific methyltransferase 2D (Kmt2d) gene on chromosome 15 (Yamamoto *et al*., 2019). Indeed, mutations with a loss of function in the KMT2D gene in humans are mainly responsible for Kabuki syndrome (Ratbi *et al*., 2013; Lu *et al*., 2016). *Bapa* mice also present hyperactivity, sensory, and psychomotor impairments, such as hypotonia, and a slight motor coordination dysfunction (Yamamoto *et al*., 2019; de Oliveira-Higa *et al*., 2023). Some of these behavioural impairments, such as hyperactivity, are also found during the prepubertal period (Kirsten *et al*., 2022). The behavioural findings were associated with dopaminergic system modulation, including increased gene expression of the D1 receptor (de Oliveira-Higa *et al*., 2023) in adults and increased striatal tyrosine hydroxylase (TH) expression in juveniles (Kirsten *et al*., 2022).

Besides our mouse model of Kabuki syndrome, there is also a model developed by Bjornsson and colleagues with a heterozygous deletion in the gene encoding the Kmt2d, leading to impairment of methyltransferase function: Kmt2d^+/βGeo^ mice (Bjornsson *et al*., 2014). This model is characterised by reduced neurogenesis, hippocampal memory defects (Bjornsson *et al*., 2014), shortened long bones and ventral bowing of skulls associated with disrupted endochondral ossification (Fahrner *et al*., 2019), and DNA methylation aberrations in peripheral blood (Goodman *et al*., 2023). There are other studies in mice that are indirectly considered experimental models of the Kabuki syndrome. For example, loss of function of one allele for the KMT2A (Mll1) and Kmt2b (Mll2) genes is also related to cognitive, hippocampal, and neurogenesis defects (Kerimoglu *et al*., 2013; Shen *et al*., 2014; Jakovcevski *et al*., 2015). There is currently no reasonable mechanism pointed out for the motor and immune deficits present in Kabuki syndrome patients.

Considering that most of the behavioural phenotype found in the Kabuki mouse model is associated with motor activity, the hypothesis of the present study is that behavioural impairments found in Kabuki syndrome are consequences of striatal dopaminergic modulation (Cerovic *et al*., 2013; Prager and Plotkin, 2019). Moreover, the immune dysfunctions found in Kabuki syndrome may be in detriment of neuroinflammatory processes, since overactivity of astrocytes and microglia and other central nervous system components may affect susceptibility to infections and immune mediators expression (Schwab *et al*., 2014; Ransohoff *et al*., 2015; Edison, 2024). To evaluate these hypotheses, the *bapa* mouse model was used. Behavioural aspects of Kabuki syndrome were evaluated, such as anxiety, exploratory behaviour, and repetitive/stereotyped behaviours. TH, the rate-limiting enzyme of the dopamine biosynthesis pathway (Baker *et al*., 2003), was studied for the dopaminergic striatal modulation hypothesis. Astrocytic glial fibrillary acidic protein (GFAP, the major protein constituent of glial intermediate filaments in differentiated astrocytes of the central nervous system) (Sofroniew and Vinters, 2010) and ionised calcium-binding adaptor molecule 1 (Iba1, a macrophage/microglial marker) (Kempuraj *et al*., 2024) were studied for the neuroinflammatory hypothesis.

## Material and methods

### Ethics statement

The authors assert that all procedures contributing to this work comply with the ethical standards of the relevant national and institutional committees on human experimentation and with the Helsinki Declaration of 1975. The authors assert that all procedures contributing to this work comply with the ethical standards of the relevant national and institutional guides on the care and use of laboratory animals.

The present study was carried out in strict accordance with the recommendations of the ARRIVE guidelines and the Guide for the Care and Use of Laboratory Animals of the National Institutes of Health. The protocol was approved by the Committee on the Ethics of Animal Experiments of the Paulista University, Brazil (Permit Number: 035/17). All efforts were made to minimise suffering, reduce the number of animals used, and utilise alternatives to *in vivo* techniques when available. The experiments were also performed in accordance with good laboratory practice protocols and quality assurance methods.

### Animals, groups, and experimental design

Female adult BALB/c and BALB/c^*bapa*^ (*bapa*) mice (*Mus musculus*) with approximately 100 days of age on the luteal phase of the oestrous cycle were used (n = 7/group). The luteal phase was confirmed through microscopic analysis of histological sections from the ovary and uterine horns (Akinjiola *et al*., 2018). Mice were obtained from the Institute of Biomedical Sciences (University of São Paulo, São Paulo, Brazil) and housed at Paulista University (São Paulo, Brazil) under standard conditions. The mice housing, nutritional conditions, and daily handling and care were standard and previously described by our group (Kirsten *et al*., 2022).

Mice were first evaluated in the light-dark test and then for spontaneous self-grooming behaviour. Twenty-four hours later, they were evaluated in the splash test, and immediately, their brains were processed for immunohistochemical analysis (TH, GFAP, and Iba1). All the experiments were performed between 9:00 and 11:00 AM to minimise the effects of circadian rhythms. All behavioural assays and the immunohistochemical analyses were performed by investigators who were blinded to the treatment groups.

### Light-dark test

The light-dark test was performed to evaluate anxiety-like behaviour and exploratory behaviour as previously described (Kirsten *et al*., 2020). This model is based on the inherent conflict between the exploratory drive to a novel place and the avoidance of a lit compartment (Campos *et al*., 2013). The apparatus consisted of an acrylic box (45 × 27 × 20 cm) containing two compartments (separated by a door with 9 × 7 cm): dark room with black walls and floor (17 cm), and a light room, with white walls and floor (26 cm) and illuminated with a white fluorescent lamp (15W, 4100K). Each mouse was individually placed in the centre of the light room, facing the wall opposite the door. The following parameters were evaluated over a period of 5 min: dark side entry latency (s), total times (s) spent in the dark and in the light sides, and total rearing frequency. The testing room, which was isolated from the experimenter, was a small room with dim lighting. A video camera mounted above the arena was used to collect the data. The apparatus was washed with a 5% alcohol/water solution before placement of the animals to obviate possible biasing effects from odour cues left by previous mice.

### Spontaneous self-grooming behaviour

Spontaneous self-grooming behaviour was evaluated immediately after a 30-min habituation period, conducted immediately after the light-dark test. Each mouse was individually placed in the centre of a clear observation cage (30 × 16 × 19 cm), and after the habituation period, the following parameters were evaluated over a period of 30 min: head washing, body grooming, paw/leg licking, and tail/genital grooming total times (s) (Kirsten and Bernardi, 2017). The testing room and cleaning procedures were the same as those used for the light-dark test. A video camera mounted in front of the arena was used to collect the data.

### Splash test

Twenty-four hours after the evaluation of the spontaneous self-grooming behaviour, the splash test was conducted in the same clear observation cage, according to a previous study (Reis-Silva *et al*., 2019). The splash test evaluated the induced self-grooming after spraying a 10% sucrose solution on the dorsal coat of each mouse. The same parameters evaluated for the spontaneous self-grooming behaviour were evaluated over a period of 5 min: head washing, body grooming, paw/leg licking, and tail/genital grooming total times (s). The testing room, video recording, and cleaning procedures were the same as those used for the spontaneous self-grooming evaluation.

### TH, GFAP, and Iba1 analyses

Immediately after behavioural tests, mice were euthanised (anaesthetic overdose, thiopental, 200 mg/kg, i.p.), and their brains were collected and fixed in 10% buffered formalin for 72 h for usual histological procedures. The striatum was studied for the immunohistochemical expression of TH, GFAP, and Iba1 proteins (Guimaraes Marques *et al*., 2019). It is considered a motor system brain area, involved in the modulation of movements, emotions, and cognition (Groenewegen, 2003), and its relation with neuroinflammation (Abg Abd Wahab *et al*., 2019; Mancini *et al*., 2021). The striatum was processed for immunohistochemical analysis as previously described (Kirsten *et al*., 2022). Briefly, four sections (5 μm thick) per rat were made from each striatum. Immunohistochemistry was performed using the chain polymer-conjugated staining method. Monoclonal anti-TH antibody (1:1000, Millipore Cat# IHCR1005-6, Chemicon IHC Select Research, Germany), polyclonal rabbit anti-GFAP immunoglobulin (1:100, Agilent Cat# Z0334, Santa Clara, CA, USA), and polyclonal rabbit anti-Iba1 immunoglobulin (1:100; GeneTex Cat# GTX101495, Irvine, CA, USA) were used as primary antibodies according to protocol from the suppliers, followed by the EnVision+ Kit for detection (Agilent Cat# K4011; HRP, Rabbit, DAB+, Santa Clara, CA, USA). Antigen retrieval was achieved by heating the slides in citrate buffer (pH 6.0) at 95^o^C for 15 min in a steamer. PBS solution instead of the primary antibody was used as the negative control during immunohistochemical staining. The sections were counterstained with Harris haematoxylin and mounted with DPX (06522, Sigma Aldrich, St Louis, MO, USA). From each individual immunostained section (for TH, GFAP, and Iba1), 5–6 photomicrographs were taken (40× objective, Nikon E200 microscope, equipped with a Nikon Coolpix digital camera linked to a liquid crystal display monitor, Kanagawa, Japan). Morphometric analysis was performed using the Image Pro-Plus 6 software (Media Cybernetics, Rockville, MD, USA), calibrated with digital colour filters regulating red, green, and blue bits, in such a way that only immunostained cells were included and the background staining was excluded from the measurement. For TH, GFAP, and Iba1 immunostaining quantification, we used the index per area to represent the extent of the area immunostained for TH in neurones, GFAP in astrocytes or Iba1 in microglial cells compared to the total area of the image (being zero, as the complete absence of staining, and one, as the total staining of the area). For statistical purposes, the means of 5–6 photomicrograph values from each mouse were used as units.

### Statistical analysis

Normality was verified using Shapiro–Wilk or Kolmogorov–Smirnov tests, depending on the sample size (alpha = 0.05). When necessary, an outlier identification test was applied (ROUT, *Q* = 5%). The Student’s *t*-test (unpaired, two-tailed) was used to compare the parametric data between the groups. The Mann–Whitney *U*-test was used to compare the nonparametric data between the groups. The results are expressed as mean ± SEM in box and whiskers (min to max, showing all values) graphs. In all cases, the results were considered significant if *p* < 0.05.

## Results

**Figure 1.**
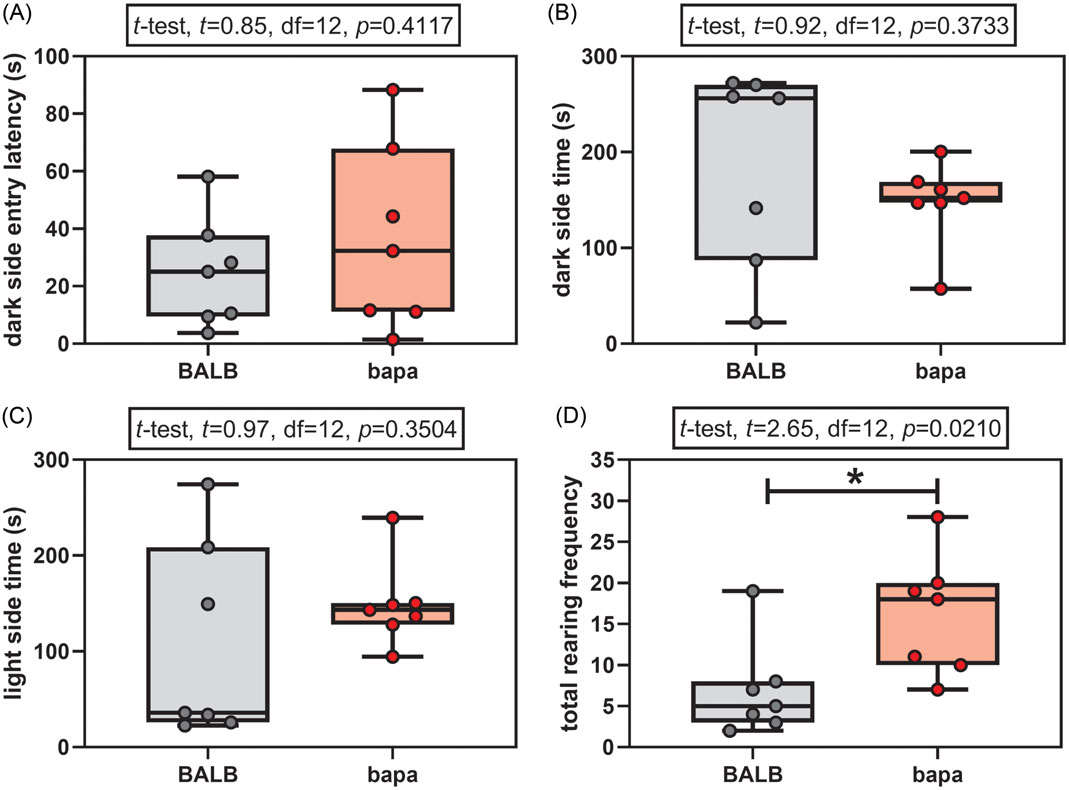
Light-dark test. Light-dark test of adult BALB/c and BALB/c^*bapa*^ (*bapa*) mice (n = 7 mice per group). *p < 0.05 (Student’s *t*-test). Data are expressed as mean ± SEM in box and whiskers (min to max, showing all values) graphs.

In the light-dark test, both groups passed the normality tests for all the evaluated parameters (Supplementary Table 1). None of the anxiety parameters was altered between groups: dark side entry latency (Fig. 1A), time spent in the dark side (Fig. 1B), and time spent in the light side (Fig. 1C). However, female *bapa* mice presented increased rearing behaviour, compared with BALB/c data (Fig. 1D). In other words, *bapa* mice did not present anxiety-like behaviour but increased exploratory behaviour.

**Figure 2.**
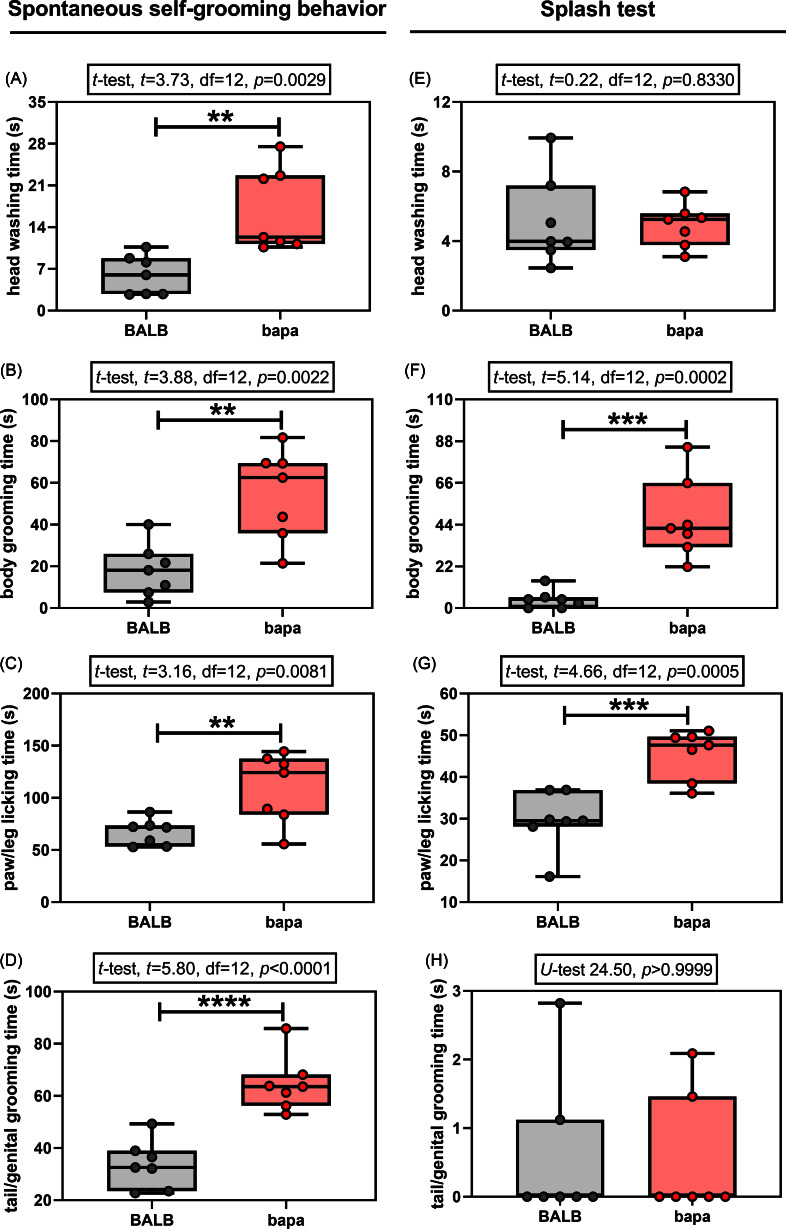
Self-grooming behaviour. Spontaneous and induced (splash test) self-grooming behaviour of adult BALB/c and BALB/c^*bapa*^ (*bapa*) mice (n = 7 mice per group). *p < 0.05; **p < 0.01; ***p < 0.001; and ****p < 0.0001 (Student’s *t*-test and Mann–Whitney *U*-test). Data are expressed as mean ± SEM in box and whiskers (min to max, showing all values) graphs.

In the spontaneous self-grooming behaviour evaluation, both groups passed the normality tests for all the evaluated parameters (Supplementary Table 1). All the evaluated parameters were altered between groups. Specifically, compared with BALB/c data, female *bapa* mice presented increased time spent for head washing (Fig. 2A), body grooming (Fig. 2B), paw/leg licking (Fig. 2C), and tail/genital grooming (Fig. 2D). Therefore, *bapa* mice presented increased spontaneous self-grooming behaviour.

In the splash test, one of the four parameters did not pass the normality tests for both groups: tail/genital grooming time (Supplementary Table 1). Compared with BALB/c data, female *bapa* mice presented increased time spent for body grooming (Fig. 2F) and paw/leg licking (Fig. 2G), but not for head washing (Fig. 2E) and tail/genital grooming (Fig. 2H). Therefore, *bapa* mice presented increased induced self-grooming behaviour in the splash test.

**Figure 3.**
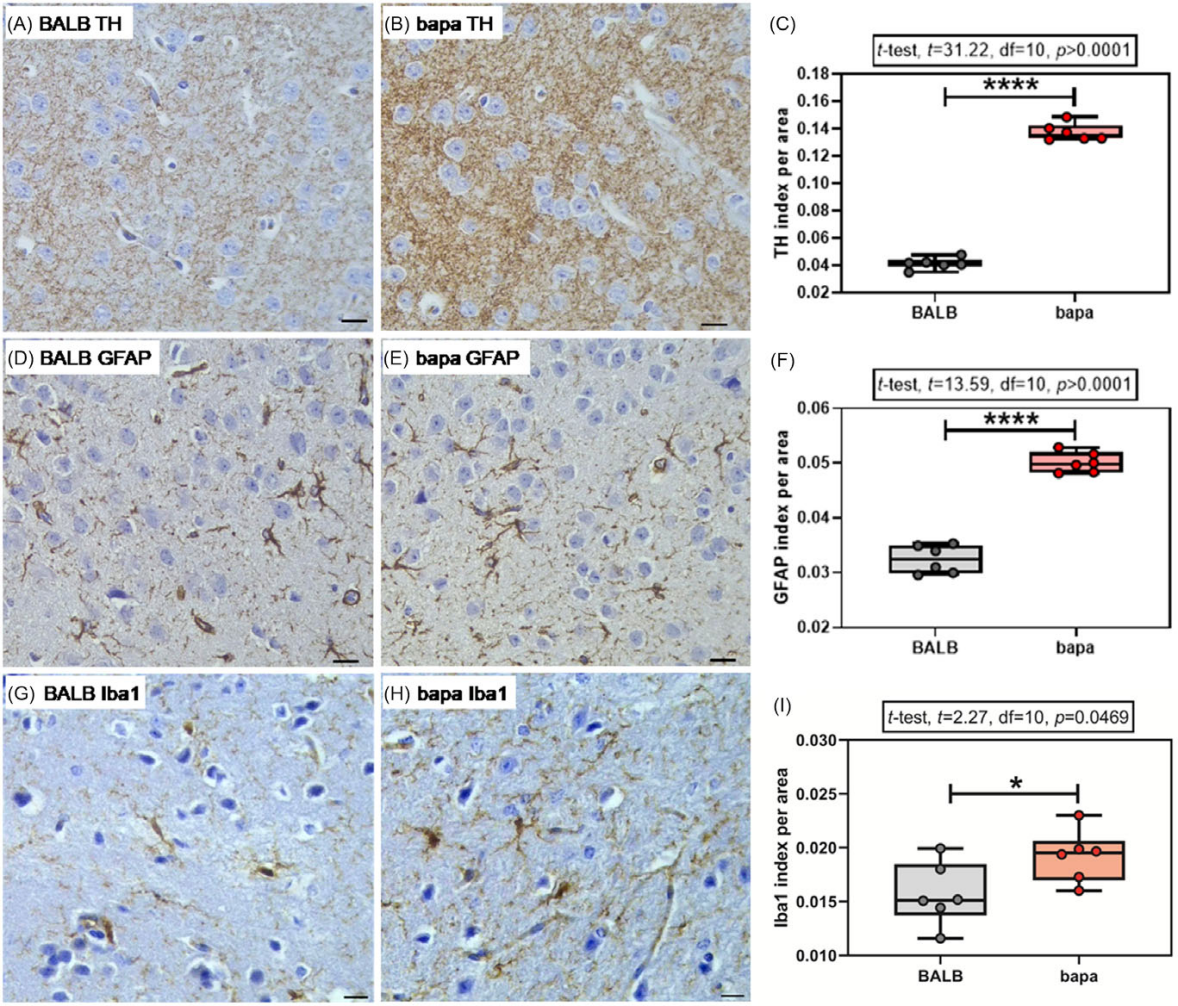
TH, GFAP, and Iba1. (A–C) Tyrosine hydroxylase (TH), (D–F) astrocyte glial fibrillary acidic protein (GFAP), and (G-I) microglial Iba1 expressions in the striatum of adult BALB/c and BALB/c^*bapa*^ (*bapa*) mice (n = 6 mice per group; 5–6 photomicrographs from each individual brain section). Morphometric analysis from TH, GFAP, and Iba1-immunolabelled sections. Scale bar = 50 μm. *p < 0.05; **p < 0.01; ***p < 0.001; and ****p < 0.0001 (Student’s *t*-test). Data are expressed as mean ± SEM in box and whiskers (min to max, showing all values) graphs.

For the immunohistochemical analysis of TH expression, both groups passed the normality tests (Supplementary Table 1). Female *bapa* mice presented increased striatal TH expression, compared with BALB/c data (Fig. 3A–C).

For the immunohistochemical analysis of GFAP expression, both groups passed the normality tests (Supplementary Table 1). Female *bapa* mice presented increased striatal GFAP expression, compared with BALB/c data (Fig. 3D–F).

For the immunohistochemical analysis of Iba1 expression, both groups passed the normality tests (Supplementary Table 1). Female *bapa* mice presented increased striatal Iba1 expression, compared with BALB/c data (Fig. 3G–I).

## Discussion

Although anxiety is not considered a core symptom of Kabuki syndrome (Van Laarhoven *et al*., 2015; Wang *et al*., 2019), there is tangible evidence that it should be considered a neurobehavioral feature: a cohort of 60 individuals with molecularly confirmed Kabuki syndrome presents higher anxiety scores than controls (Kalinousky *et al*., 2022). Moreover, patients with a mosaic KMT2D variant were described with similar systemic features and anxiety manifestations (Boniel *et al*., 2021). Thus, the present study evaluated anxiety-like behaviour in the *bapa* mouse model. The light-dark test was chosen for the evaluation of anxiety-like behaviour since it is considered a reliable and popular test for this purpose on mice (Bourin and Hascoet, 2003; Campos *et al*., 2013).

Presently, none of the evaluated anxiety parameters were affected; that is, female adult *bapa* mice did not present anxiety-like behaviour. Similarly, prepubertal and pubertal *bapa* mice do not present anxiety-like behaviour evaluated on the open-field test (Kirsten *et al*., 2022). Adult *bapa* mice also do not present anxiety-like behaviour evaluated on the elevated plus maze test (Oliveira, 2017). Therefore, although anxiety is a secondary symptom of Kabuki syndrome, the *bapa* mouse model consistently did not result in anxiety-like behaviour.

However, female *bapa* mice presented increased exploratory behaviour demonstrated by increased rearing frequency in the light-dark test. This pattern was previously demonstrated in the *bapa* mouse model using different variables: adult males and females in the open-field test (increased distance travelled, average speed, and rearing) (Yamamoto *et al*., 2019); adult males in the open-field test observed for four consecutive days (increased locomotion and rearing and decreased immobility) (de Oliveira-Higa *et al*., 2023); and prepubertal period in the open-field test (increased rearing frequency) (Kirsten *et al*., 2022), always comparing to their controls. Thereby, *bapa* mice presented locomotory hyperactivity, a symptom described in patients with Kabuki syndrome (Mervis *et al*., 2005; Sertcelik *et al*., 2016).

To understand the neurobiological mechanism responsible for the locomotory hyperactivity, striatal TH expression was evaluated. Female adult *bapa* mice presented increased striatal TH expression, indicating increased dopamine synthesis in the striatal dopaminergic system (Baker *et al*., 2003). A similar result was found in the prepubertal period (Kirsten *et al*., 2022). Additionally, adult male *bapa* mice present increased gene expression of the striatal D1 receptor (de Oliveira-Higa *et al*., 2023). Taken together, the behavioural findings were associated with dopaminergic system modulation; that is, *bapa* mice presented striatal dopaminergic system hyperactivity, which triggered an increased exploratory activity.

Self-grooming of mice was evaluated because it is a behavioural tool used for studies about stereotyped behaviour (Crawley, 2012), which is reported in some patients with Kabuki syndrome (Sertcelik *et al*., 2016; Boniel *et al*., 2021). Self-grooming is considered a behavioural pattern very sensitive to genetic manipulations and to study genotype differences among selected mouse strains (Kalueff *et al*., 2007) and human neurological disorders (Kalueff *et al*., 2016). Therefore, the self-grooming behavioural study was applied for the understanding of the mutant mouse *bapa* and the Kabuki syndrome.

A typical grooming chain in rodents is embedded into different predictable patterns and microstructures, which include head washing, body grooming, paw/leg licking, and tail/genital grooming (Kalueff *et al*., 2007). Simply assessing the ‘amount’ of animal total grooming may be insufficient for correct data interpretation and analysis (Kalueff *et al*., 2007). Self-grooming can be elicited by various environmental conditions, such as under basal conditions, after an acclimation period in the recording chamber and induced artificially, for example, following misting rodents with water (using spray) (Kalueff *et al*., 2007). The present study evaluated these two environmental conditions: basal for the spontaneous grooming and the elicited grooming (using a spray – splash test). Both models revealed increased self-grooming in the female *bapa* mice, compared with the controls. However, the spontaneous self-grooming test resulted in more expressive differences than the splash test. Taken together with its natural conception and a less stressful protocol, the spontaneous self-grooming test was considered a reliable test to study the mutant mouse *bapa* and the Kabuki syndrome.

Self-grooming sequencing, chain initiation, and chain completion in rodents are strongly bidirectionally affected by striatal dopaminergic system modulation, including lesions of the dopamine-containing nigrostriatal tract, administration of various dopaminergic drugs, genetic mutations, and psychological stress (Cromwell and Berridge, 1996; Burguiere *et al*., 2013; Kalueff *et al*., 2016). For example, dopamine D1 receptor activation by D1 agonists induces excessive grooming (Berridge and Aldridge, 2000). Similarly, transgenic mice with reduced TH immunoreactivity in different brain areas present impaired grooming behaviour (Aloe and Fiore, 1997). Thus, the increased TH expression in the striatum of *bapa* mice was hypothesised as the explanation of the pathway responsible for the increased self-grooming and stereotyped behaviour. However, further molecular studies should be conducted to reveal if this is the only pathway involved and to deepen the knowledge.

As previously reported, Kabuki syndrome patients are commonly diagnosed with immune system deficiencies. It is described as a lack of antibodies, loss of memory cells, IgA deficiency, hyper-IgM syndrome, disturbed differentiation of terminal B-cells (humoral immunodeficiency), and some autoimmune diseases (Van Laarhoven *et al*., 2015; Wang *et al*., 2019). The hypothesis of the present study was that this immune dysfunction may be in detriment of neuroinflammation, since overactivity of astrocytes and microglia, as well as other central nervous system components may affect susceptibility to infections and immune mediators’ expression (Schwab *et al*., 2014; Ransohoff *et al*., 2015; Edison, 2024). As Kabuki syndrome is characterised by immune dysregulation, up to 17% of its patients present immune thrombocytopenia, often associated with other hematological autoimmune diseases, including autoimmune haemolytic anaemia, eventually resulting in Evans disease, with recurrent respiratory diseases and chronic lung inflammation (Leonardi *et al*., 2023). In fact, *bapa* mice presented increased striatal GFAP and Iba1 expressions. Clearing of dopamine excess may also explain astrocyte hyperactivity since these cells perform metabolic, structural, homeostatic, and neuroprotective functions (Sofroniew and Vinters, 2010). It is recognised that astrocytes from the striatum and cortex express D1-like (D1 and D5) and D2-like receptors (D2, D3, and D4) and dopamine signalling impacts on astrocyte morphology and gene expression (Corkrum and Araque, 2021). Inflammation induced by activated microglia can directly damage dopaminergic neurones, inhibiting dopamine synthesis, reuptake, and receptor activity (She *et al*., 2024). Thereby, the increased expression of striatal GFAP and Iba1 revealed a neuroinflammatory process, which was hypothesised as the responsible for the immune dysfunction found in the Kabuki syndrome.

It is important to mention that the increased striatal GFAP and Iba1 expressions were observed in female adult *bapa* mice not challenged with any immunological agent, which reassembled not an acute, but a permanent (chronic) striatal neuroinflammatory process. This is a novel result, considering that the previous study of our group revealed increased GFAP expression in juveniles after an LPS challenge (Kirsten *et al*., 2022).

Incidentally, a persistent acute neuroinflammation can turn to a chronic neuroinflammation as it accumulates damage, resulting in neuronal degeneration (Abg Abd Wahab *et al*., 2019). Neuroinflammation is the response of the central nervous system to disturbed homeostasis and typifies several neurological and neurodegenerative diseases, such as Parkinson’s disease, Huntington’s disease, multiple sclerosis, narcolepsy, and autism (Ransohoff *et al*., 2015; Abg Abd Wahab *et al*., 2019; Mancini *et al*., 2021). Specifically, astroglial-mediated inflammation plays a prominent role in the pathogenesis of neurodegenerative diseases, such as dementia and Alzheimer’s disease (Edison, 2024). In fact, intracellular signalling pathways are completely controlled by astrocytes during inflammation. Astrocytes and microglia are involved in cellular and molecular functions for degeneration, vascular signalling, and glial–neuronal interactions (Abg Abd Wahab *et al*., 2019).

Besides the explanation of the peripheral immunological deficits found in the Kabuki syndrome, striatal neuroinflammation could also explain the striatal dopaminergic system hyperactivity (behaviour and TH expression) presently found. There is robust evidence of the cross talk between neurotransmitters and neuroinflammation in the striatum in the mediation of motor behaviour (Abg Abd Wahab *et al*., 2019; Mancini *et al*., 2021). For example, neuroinflammation is found to be involved in the alterations in dopamine neurotransmission, whereby cytokines ultimately lead to decreased dopamine synthesis, thus decreasing dopamine function, which could lead to neurodegeneration (Abg Abd Wahab *et al*., 2019). Apropos, dopamine can act as both an inhibitory and excitatory neurotransmitter depending upon its location in the brain and which receptor it binds to (Nakamura *et al*., 2014). Moreover, the striatum acts as one of the main target regions for dopamine involving the regulation of motor functions (Abg Abd Wahab *et al*., 2019). Therefore, it is plausible that the striatal dopaminergic system hyperactivity in the *bapa* mice is a consequence of a permanent striatal neuroinflammatory process.

The present study evaluated only female mice based on our previous studies. The motor/exploratory hyperactivity of adult *bapa* mice found in the behavioural assessments (increased distance travelled, average speed, and rearing) presents the same statistical difference, comparing male and female *bapa* mice with their respective controls (Yamamoto *et al*., 2019). The same scenario is revealed in the behavioural and brain analyses of prepubertal and pubertal mice (Kirsten *et al*., 2022). Therefore, considering that there are no sex-specific effects in the behavioural and brain tests of *bapa* mice, the effort to minimise the number of animals used in the experiments, and the tendency in neuroscience studies to avoid sex-discrimination (comparing males and females), only female mice were studied in the present study

In conclusion, *bapa* mice did not present anxiety-like behaviour but exploratory hyperactivity and stereotyped behaviour. This phenotype occurred in detriment of the striatal dopaminergic system hyperactivity and a permanent neuroinflammatory process.

## Supporting information

Biondi et al. supplementary material Biondi et al. supplementary material

## Data Availability

All data underlying the findings described in the manuscript are fully available without restriction. Data can be accessed by contacting the corresponding author. All relevant data are within the paper and its Supporting Information files.

## Supplementary material

The supplementary material for this article can be found at https://doi.org/10.1017/neu.2025.17.
