# Supplementary material for: Behavioural impairments in a mouse model of Kabuki syndrome associated with dopaminergic and neuroinflammatory modulations
Source: Acta Neuropsychiatr. 2025 Apr 14;37:e63. doi: 10.1017/neu.2025.17 (PMC13130331; doi:10.1017/neu.2025.17)
Supplement: Biondi et al. supplementary material [file S0924270825000171sup001.pdf]

Title: Behavioral impairments in a mouse model of Kabuki syndrome associated with dopaminergic and neuroinflammatory modulations

Supplementary Table 1. Normality tests. Normality tests values using Shapiro-Wilk (W) test or Kolmogorov-Smirnov (KS) test for light-dark test, spontaneous self-grooming behavior, splash test, tyrosine hydroxylase (TH), astrocyte glial fibrillary acidic protein (GFAP), and ionized calcium-binding adaptor molecule 1 (Iba1) expressions in the striatum of adult BALB/c and BALB/c^{bapa} (*bapa*) mice (n=6-7 mice per group)

**Parameter / Group**	**Passed normality test?**	BALB	*bapa*
Light-dark test			
dark side entry latency	yes	KS=0.1995, *p*>0.1000	KS=0.2100, *p*>0.1000
dark side time	yes	W=0.8203, *p*=0.0646	W=0.8239, *p*=0.0700
light side time	yes	W=0.8132, *p*=0.0552	W=0.8290, *p*=0.0783
total rearing frequency	yes	KS=0.2785, *p*>0.1000	KS=0.1897, *p*>0.1000
Spontaneous self-grooming behavior			
head washing time	yes	W=0.8650, *p*=0.1676	W=0.8143, *p*=0.0566
body grooming time	yes	W=0.9628, *p*=0.8427	W=0.9404, *p*=0.6422
paw/leg licking time	yes	W=0.9074, *p*=0.3784	W=0.8984, *p*=0.3215
tail/genital grooming time	yes	W=0.9416, *p*=0.6529	W=0.8779, *p*=0.2173
Splash test			
head washing time	yes	W=0.8847, *p*=0.2480	W=0.9749, *p*=0.9315
body grooming time	yes	W=0.8400, *p*=0.0993	W=0.9182, *p*=0.4552
paw/leg licking time	yes	W=0.8446, *p*=0.1096	W=0.8299, *p*=0.0799
tail/genital grooming time	no	W=0.6289, *p*=0.0006***	W=0.6477, *p*=0.0010***
Striatal TH expression	yes	KS=0.2392, *p*>0.1000	KS=0.2544, *p*>0.1000
Striatal GFAP expression	yes	KS=0.2246, *p*>0.1000	KS=0.1875, *p*>0.1000
Striatal Iba1 expression	yes	KS=0.2383, *p*>0.1000	KS=0.2260, *p*>0.1000

**p*<0.05; ***p*<0.01; and ****p*<0.001
